# Effect of Sitafloxacin and Amikacin Liposome Inhalation Suspension on Clarithromycin-Resistant Pulmonary Mycobacterium avium Complex Disease: A Case Series

**DOI:** 10.7759/cureus.80607

**Published:** 2025-03-15

**Authors:** Yuki Yamazaki, Masashi Matsuyama, Hiroya Sunabe, Chio Sakai, Nobuyuki Hizawa

**Affiliations:** 1 Department of Respiratory Medicine, University of Tsukuba, Ibaraki, JPN

**Keywords:** 65 years old, amikacin liposome inhalation suspension (alis), cavitary lesions, clarithromycin (cam)-resistant pulmonary mycobacterium avium complex (mac) disease, sitafloxacin (stfx)

## Abstract

Clarithromycin (CAM)-resistant pulmonary *Mycobacterium avium *complex (MAC) disease poses significant treatment challenges. This case series describes four patients treated with sitafloxacin (STFX) and amikacin liposome inhalation suspension (ALIS) in addition to standard guideline-based therapy (GBT). Sputum culture negativity was achieved in two patients, both under 65 years old and without cavitary lesions. These findings suggest the potential efficacy of STFX and ALIS as adjuncts to GBT for CAM-resistant pulmonary MAC disease, particularly in younger patients with non-cavitary disease.

## Introduction

Clarithromycin (CAM)-resistant pulmonary *Mycobacterium avium* complex (MAC) disease is a refractory infectious lung disease with limited treatment options [1]. The 2020 international guideline recommends at least two to three months of parenteral aminoglycoside administration in a three-drug, macrolide-based regimen (guideline-based therapy (GBT)) for CAM-resistant pulmonary MAC disease [2]. The international guideline also suggests seeking expert consultation.

Amikacin liposome inhalation suspension (ALIS) has recently emerged as a treatment for refractory pulmonary MAC disease [3]. For CAM-resistant pulmonary MAC disease, the addition of ALIS to GBT has demonstrated a culture conversion rate of 13.7% within 6 months (compared with 4.5% for GBT alone) [3]. However, real-world reports of GBT + ALIS for refractory pulmonary MAC disease indicate that this combination has limited efficacy against CAM-resistant pulmonary MAC disease [4,5].

The international guideline notes that fluoroquinolones are not recommended for treating pulmonary MAC disease due to insufficient evidence from studies with small sample sizes [2]. Nonetheless, a sitafloxacin (STFX)-based regimen has shown promise for CAM-resistant pulmonary MAC disease, as reported in Japan [6].

Although STFX and ALIS may each be effective for CAM-resistant pulmonary MAC disease, the impact of combining these treatments remains unclear. This case series evaluated the outcomes of combining STFX and ALIS with GBT in four patients with CAM-resistant pulmonary MAC disease. Two patients achieved sputum culture negativity after six months, and both were under 65 years of age and had no cavitary lesions.

## Case presentation

Case 1

**Figure 1:**
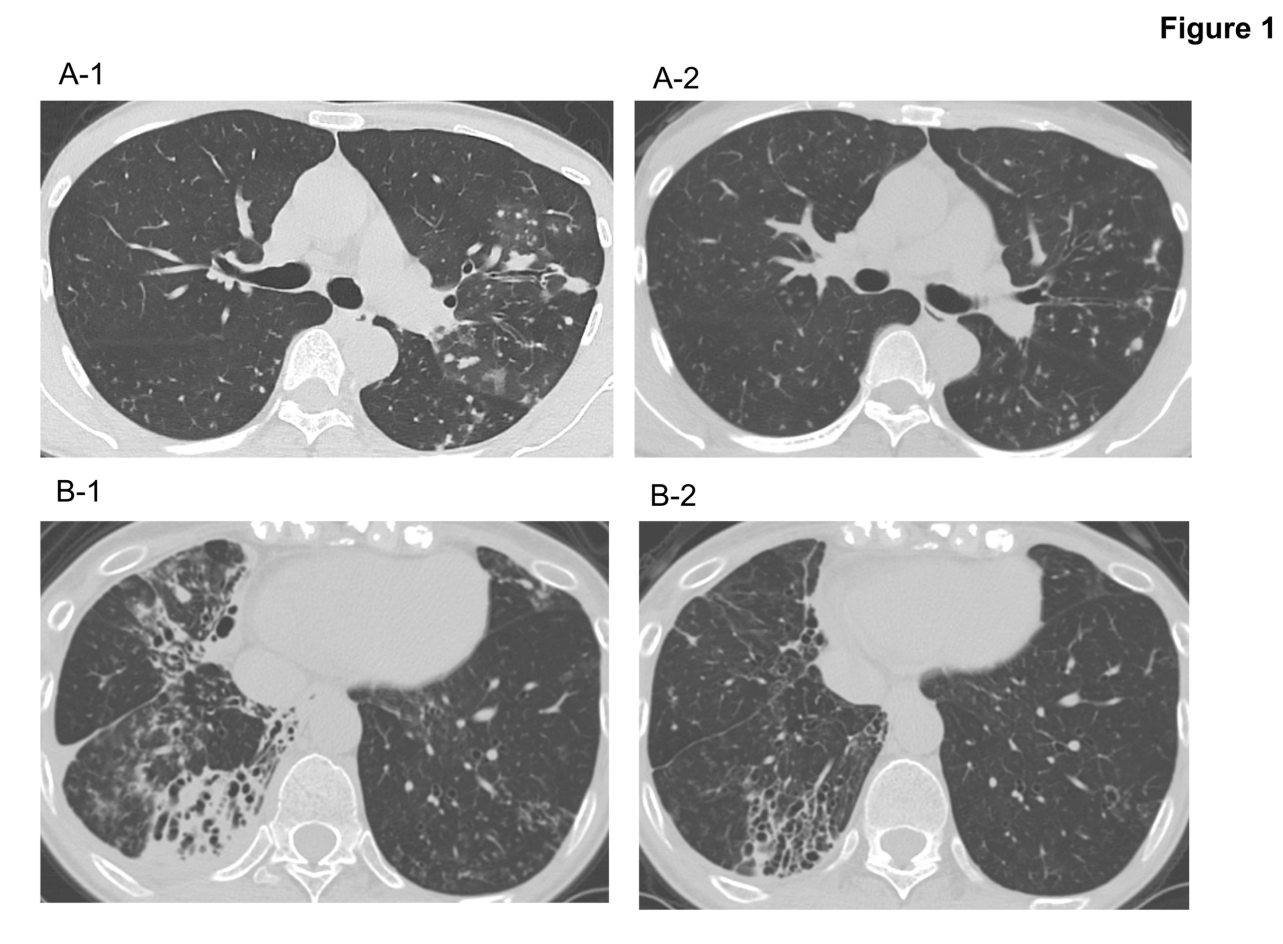
Chest CT before and after administration of STFX and ALIS in Cases 1 (responder) and 2 (responder) of CAM-resistant pulmonary MAC disease (A-1) Chest CT before STFX+ALIS in case 1. (A-2) Chest CT after STFX+ALIS in case 1. (B-1) Chest CT before STFX+ALIS in case 2. (B-2) Chest CT after STFX+ALIS in case 2. Abbreviations STFX: sitafloxacin, ALIS: amikacin liposome inhalation suspension, CAM: clarithromycin, MAC: *Mycobacterium avium* complex

This case involved a 43-year-old woman with a history of allergic bronchopulmonary aspergillosis, in whom STFX and ALIS were added to GBT. She had no history of smoking but had a family history of pulmonary MAC disease, with her mother and sisters also affected. She initially presented with worsening cough and sputum production. The patient had been diagnosed with pulmonary MAC disease in 2010 and was treated with CAM 800 mg/day, ethambutol (EB) 500 mg/day, and rifampicin (RFP) 450 mg/day. Due to pregnancy, she discontinued treatment from 2015 to 2017 but resumed thereafter. In 2021, she was referred to our hospital due to worsening cough and dyspnea. Chest computed tomography (CT) showed no obvious cavitary shadows but showed diffuse small nodular shadows (Figure 1, panel A-1). Resistance to CAM was confirmed, and CAM was substituted for azithromycin (AZM) 250 mg/day to improve medication compliance. Three months later, STFX 200 mg/day and ALIS 590 mg/day were introduced to her treatment regimen. After five months of combined treatment, her sputum culture became negative, and chest CT showed significant improvement (Figure 1, panel A-2). Due to dysphonia, ALIS administration was adjusted to every other day at seven months. At 31 months, all medications except erythromycin (EM) were successfully discontinued.

Case 2

This case involved a 55-year-old woman who received STFX and ALIS as part of her treatment regimen for pulmonary MAC disease. She had a history of gastric ulcers but had never smoked. She was diagnosed with pulmonary MAC disease in 2019. Initial treatment with AZM 250 mg/day, EB 750 mg/day, and RFP 450 mg/day failed to achieve sputum culture conversion. CAM resistance was detected in 2022. Chest CT showed bronchiectasis and infiltrative shadows localized to the right middle and lower lobes (Figure 1, panel B-1), but no obvious cavities were observed. Due to radiological worsening and persistent productive cough, STFX 200 mg/day was added in 2023. Four months later, ALIS 590 mg/day was introduced. Within a week, the patient developed dysphonia, necessitating a temporary withdrawal of ALIS for two weeks. Upon resumption at an adjusted dosing schedule of every other day, sputum culture negativity was achieved within two months, with notable radiological improvement (Figure 1, panel B-2). The patient remained culture-negative for 16 months, demonstrating sustained microbiological and radiological improvement with the addition of STFX and ALIS to her treatment regimen.

Case 3

**Figure 2:**
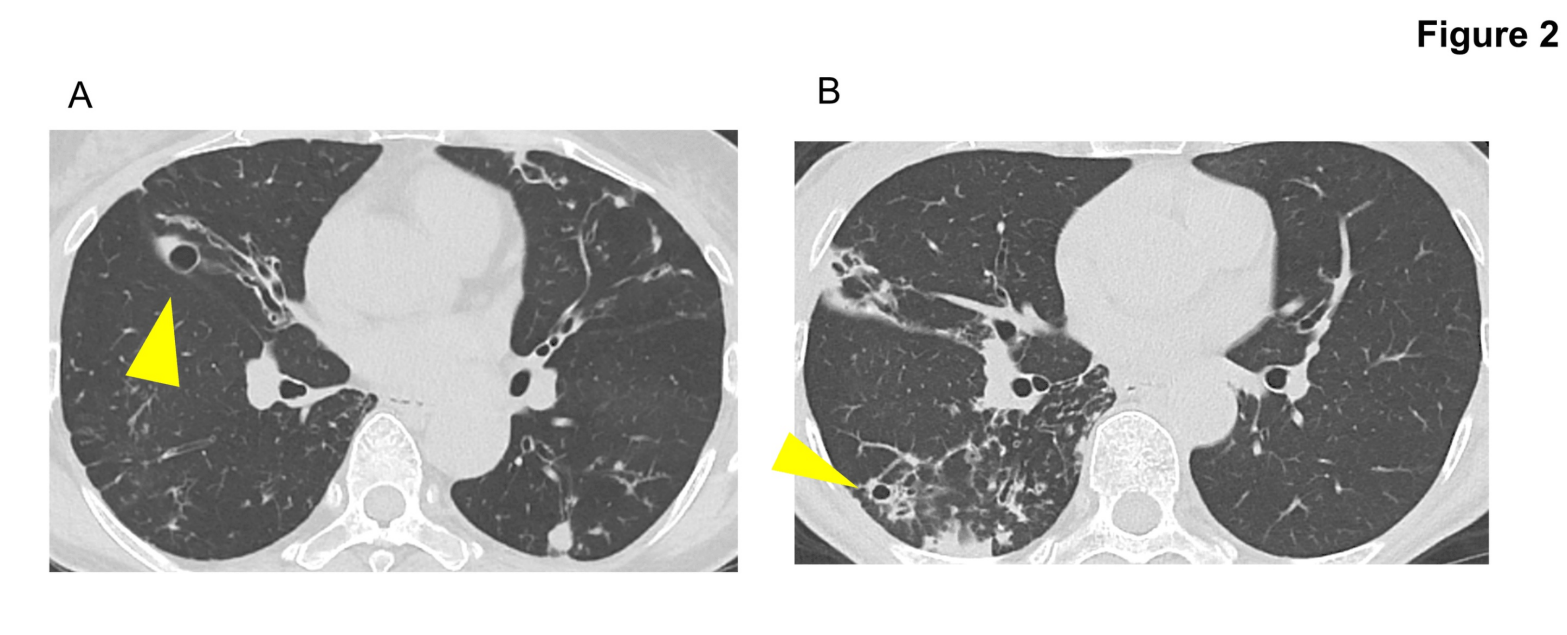
Chest CT before the administration of STFX and ALIS in cases 3 (non-responder) and 4 (non-responder) of CAM-resistant pulmonary MAC disease (A) Chest CT before STFX+ALIS in case 3. The arrow shows the cavity. (B) Chest CT before STFX+ALIS in case 4. The arrow shows the cavity. Abbreviations STFX: sitafloxacin, ALIS: amikacin liposome inhalation suspension, CAM: clarithromycin, MAC: *Mycobacterium avium* complex

This case involved a 62-year-old woman with a history of asthma and hypertension, who received STFX and ALIS as part of her treatment for pulmonary MAC disease. She had never smoked. The patient was initially diagnosed with pulmonary MAC disease in 1997. Early treatment consisted of CAM monotherapy, which was subsequently discontinued. Combination therapy with CAM, EB, and RFP was later initiated. In 2014, cavitary lesions were detected on imaging, and CAM resistance was identified. However, the patient was unable to adhere to long-term treatment. In 2023, she was referred to our hospital with worsening cavitary lesions (Figure 2, panel A). Given her disease progression, treatment was initiated with EM 400 mg/day, RFP 450 mg/day, EB 750 mg/day, STFX100mg/day, and ALIS 590 mg/day. Due to dysphonia, ALIS was initially administered every other day for one month before transitioning to daily dosing. Despite eight months of treatment, sputum cultures remained positive.

Case 4

This case involved a 75-year-old woman with dyslipidemia who was treated for pulmonary MAC disease and bronchiectasis. She was diagnosed with bronchiectasis in 2006 and initially received CAM 200 mg/day monotherapy. In 2017, pulmonary MAC disease was diagnosed, and she started combination therapy with CAM 800 mg/day, EB 500 mg/day, and RFP 300 mg/day. In 2021, AZM 250 mg/day replaced CAM 800mg/day to improve medication adherence, and STFX 100 mg/day was added due to a worsening of infiltrative shadows and cavity formation (Figure 2, panel B). CAM resistance was confirmed in 2023, and she was referred to our hospital. AZM 250 mg/day was switched to EM 400 mg/day, and the STFX dose was increased to 200 mg/day. ALIS 590 mg/day was introduced six months later. Two weeks after starting ALIS, dysphonia developed, leading to a dose adjustment to every other day. Despite eight months of treatment, sputum cultures remained positive, indicating persistent microbiological activity.

Four cases of CAM-resistant pulmonary MAC disease treated with GBT supplemented with STFX and ALIS were described. The clinical and radiological characteristics of these cases are summarized in Table 1. The extent of pulmonary disease was assessed using previously reported CT scoring methods [7]. The locations of the lesions and the number of involved lobes were analyzed. The lesion locations were evaluated in separate sections, that is, the upper, middle (lingula), and lower lobes, with the lingula considered a separate lobe. The number of lobes with lesions was defined as the CT score.

**Table 1:** Comparison of four cases with clarithromycin (CAM)-resistant pulmonary Mycobacterium avium complex (MAC) disease treated with sitafloxacin (STFX) plus amikacin liposome inhalation suspension (ALIS) over standard guideline-based therapy (GBT) Abbreviations *M. avium*: *Mycobacterium avium*, BMI: body mass index, ESR: erythrocyte sedimentation rate, BACES: body mass index, age, cavity, erythrocyte sedimentation rate, and sex, STFX: sitafloxacin, ALIS: amikacin liposome inhalation suspension, EM: erythromycin, AZM: azithromycin, GBT: guideline-based therapy

Case	Age	Sex	Species	BMI before STFX+ALIS	Cavity/diameter before STFX+ALIS (mm)	ESR before STFX+ALIS (mm/hr)	BACES score before STFX+ALIS	CRP before STFX+ALIS (mg/dL)	GBT before STFX+ALIS	Treatment in addition to STFX+ALIS	Total treatment period before STFX+ALIS (Years)	CT score before STFX+ALIS	Culture negative at 6 months
1	43	Female	M. avium	16.2	-	9	1	0.03	AZM+EB+RFP	AZM+EB+RFP	11	6	+
2	55	Female	M. avium	18.5	-	5	0	0.06	AZM+EB+RFP	AZM+EB+RFP	2	3	+
3	62	Female	M. avium	23	13	24	2	0.12	No medication	EM+EB+RFP	10	5	-
4	75	Female	M. avium	16.4	8	10	3	0.03	AZM+EB+RFP	EM+EB+RFP	7	3	-

## Discussion

The prognosis for CAM-resistant pulmonary MAC disease remains poor, with a reported 5-year survival rate of 53% and a sputum culture-negative rate of only 15% [1]. Recent studies have highlighted the potential of STFX and ALIS to enhance sputum culture conversion in CAM-resistant cases [3,6]. Notably, a recent case report described a patient with refractory pulmonary MAC disease who had a significant clinical response to STFX, despite the failure of both GBT and subsequent ALIS treatment [8]. While this report suggests that STFX may be effective in certain patients with pulmonary MAC disease, it is important to note that this drug is not yet widely recognized as a standard treatment option in international guidelines.

Side effects of ALIS include dysphonia, and side effects of STFX include hepatic dysfunction and QT prolongation. Dysphonia was observed in all four cases, but all showed improvement in dysphonia with every other day administration of ALIS. Hepatic dysfunction and QT prolongation were not observed in the four cases.

In cases 3 and 4, EM was used for its immunomodulatory effect, not as an antibiotic [2]. It was reported that EM monotherapy for pulmonary MAC disease may not induce cross-resistance to clarithromycin [9]. Moreover, it has also been suggested that patients with CAM-resistant pulmonary MAC disease may change to CAM-susceptible after discontinuing CAM/AZM or switching to EM [10]. Therefore, EM was used in cases 3 and 4 with the expectation of an immunomodulatory effect and a change in CAM susceptibility. 

In our case series, two patients achieved sputum culture negativity, supporting the combined use of STFX and ALIS as an adjunct to GBT. This therapy may serve as an alternative to parenteral aminoglycosides and surgical interventions, which are currently regarded as the most effective options for this condition [11]. Notably, the two patients who achieved sputum culture negativity were under 65 years of age and lacked cavitary lesions, findings consistent with the prognostic implications of BACES scoring [12]. The presence of cavitary lesions, which signify a higher bacterial burden, and older age, associated with increased susceptibility, likely contributed to the poorer outcome in the other two cases [13,14]. Cases 1 (responder) and 3 (non-responder) received STFX and ALIS simultaneously to minimize the risk of drug resistance, whereas cases 2 (responder) and 4 (non-responder) received STFX followed by sequential ALIS. These findings suggest that treatment efficacy was more closely related to the presence or absence of cavitary lesions and patient age rather than the timing of STFX and ALIS administration.

## Conclusions

The present findings suggest that the combination of STFX and ALIS may hold particular promise for younger patients with non-cavitary CAM-resistant pulmonary MAC disease. However, further studies involving larger cohorts are necessary to confirm these observations and define the optimal patient population for this treatment strategy.
